# Improvement influenza vaccine immune responses with traditional Chinese medicine and its active ingredients

**DOI:** 10.3389/fmicb.2023.1111886

**Published:** 2023-03-07

**Authors:** Danping Zhao, Xiuhong Chen, Linyuan Wang, Jianjun Zhang, Ruilin Lv, Lingyun Tan, Yawen Chen, Ran Tao, Xinyu Li, Yan Chen, Wei He, Jing He

**Affiliations:** ^1^School of Chinese Materia Medica, Beijing University of Chinese Medicine, Beijing, China; ^2^School of Traditional Chinese Medicine, Beijing University of Chinese Medicine, Beijing, China; ^3^Department of Immunology, School of Basic Medical Sciences, Anhui Medical University, Hefei, China

**Keywords:** influenza vaccine, traditional Chinese medicine, active ingredients, adjuvant, immune responses

## Abstract

The current influenza vaccines are unable to provide effective protection in many cases, like influenza viruses strain antigenic drift or shift, and the influenza continues to cause significant annual morbidity and mortality. Improving the immune response to influenza vaccination is an unmet need. Traditional Chinese medicine (TCM) and its active ingredients are commonly known to have immunomodulatory properties. We therefore compared influenza vaccination alone or formulated with Astragali Radix (Huangqi in Chinese), and several representative ingredients of TCM, including lentinan (polysaccharide), panax notoginseng saponins (saponin), breviscapine (flavone), andrographolide (terpenoid), and a Chinese herbal compound (kangai) for their potential to enhance immune responses to influenza vaccine in mice. We found that all these TCM-adjuvants were able to increase hemagglutination inhibition (HAI) antibody titers, splenocyte proliferation, splenic T cell differentiation, bone marrow dendritic cell maturity, and both Th1 and Th2 cytokine secretion of influenza vaccine to varying degrees, and that had the characteristics of no excessive inflammatory responses and bidirectional regulation simultaneously. Taken together, our findings show that Astragali Radix exerts a more comprehensive effect on vaccine immunity, on both innate and adaptive immunity. The effects of lentinan and andrographolide on adaptive immunity were more significant, while the effects of breviscapine on innate immunity were stronger, and the other two TCM adjuvants were weaker. As the first report of a comprehensive evaluation of TCM adjuvants in influenza vaccines, the results suggest that TCM and their active ingredients are good candidates for enhancing the immune response of influenza vaccines, and that suitable TCMs can be selected based on the adjuvant requirements of different vaccines.

## 1. Introduction

Influenza is a highly infectious human respiratory disease that can cause pandemics or seasonal epidemics. An estimate of country-specific influenza-associated respiratory mortality for 33 countries reported that 291,243–645,832 deaths (4.0–8.8 per 100,000 individuals) occur annually (Iuliano et al., 2018). Influenza-associated respiratory mortality in mainland China was estimated per 100,000 person seasons at 1.5 (95% CI 1.1–1.9) for individuals younger than 60 years and 38.5 (36.8–40.2) for individuals aged 60 years or older (Li et al., 2019). Although vaccination is the most effective way to prevent the development of influenza, current influenza vaccines do not provide effective protection in many cases, like antigenic drift and shift in influenza viruses (Wang W. C. et al., 2022). To match the antigenicity of the circulating viruses, vaccine seed viruses must be replaced periodically, which also leads to time-consuming surveillance and low vaccine (Yamayoshi and Kawaoka, 2019). To overcome these vaccine shortages, adjuvants have been used to improve the immune response to influenza vaccine, such as aluminum salts, MF59, and AS03. However, these adjuvants have the disadvantages of having limited approval for use in humans and failing to induce cytotoxic T cell response, and are ineffective with weak antigens, cause systemic symptoms and so on (Zhu et al., 2021). Therefore, there is an unmet need to improve the immune response to influenza vaccines.

Traditional Chinese medicine (TCM) has been practiced in China for millennia and most of them have immunomodulatory properties (Zhang et al., 2020). The World Health Organization recognizes TCM and has included TCM in the International Statistical Classification of Diseases and Related Health Problems (ICD-11; Lam et al., 2019). TCM is multicomponent medicine, which includes polysaccharides, saponins, flavonoids, terpenoids, and so on. Due to the significant effects, wide range of sources, and high safety of TCM, several studies have described their application as vaccine adjuvants or immunomodulators. TCM polysaccharides have a significant immunomodulatory effect, gaining increased attention on vaccine adjuvants, such as *Astragalus* polysaccharides, *Ganoderma lucidum* polysaccharides, *Lycium barbarum* polysaccharides, *Angelica sinensis* polysaccharides, *Codonopsis pilosula* polysaccharides, *Artemisia rupestris L.* polysaccharides, *Cistanche deserticola* polysaccharides, *Pinus massoniana pollen* polysaccharides, *Ginseng* polysaccharide, and *Eucommia ulmoides* polysaccharide, have been reported to have adjuvant effect on influenza, hepatitis B virus, foot-and-mouth disease virus, Newcastle disease virus, and on other human or veterinary vaccines (Wang et al., 2021; Wan et al., 2022). Another candidate adjuvant of TCM is saponins. Saponins from Panax ginseng, *Astragalus species*, *Panax notoginseng*, *Cochinchina momordica*, *Glycyrrhiza uralensis*, and *Achyranthes bidentata* have been reported to trigger innate immunity to exert their adjuvant activities (Song and Hu, 2009). QS-21 extracted from the bark of *Quillaja saponaria* Molina has been approved for a series of commercial vaccines as an adjuvant, and QS-21-containing combination adjuvants have also been extensively studied (Wang, 2021). Flavonoids affect the immune system response and might have immune modulator effects (Hosseinzade et al., 2019). Studies have shown that propolis flavonoid used as an adjuvant for the porcine parvovirus vaccine (Ma et al., 2022) and inactivated avian influenza and Newcastle disease vaccine (Fan et al., 2012) could improve the immune responses. Terpenoids have also been shown to have efficacy as vaccine adjuvants efficacy (Tiwari et al., 2018). Phytol derivatives induce and persist a specific immune response as an adjuvant of vaccine formulations (Aachoui et al., 2011). Squalene is a natural triterpene founded in certain fish liver oils, squalene-based emulsion adjuvants such as MF59, AS03, and AF03, have been registered for administration with influenza vaccines (Nguyen-Contant et al., 2021). However, the mechanism of adjuvanticity of TCM remains largely unknown despite its widespread use in a variety of experimental vaccines, valuable insights have been gained through ongoing research. This can regulate the immune system by promoting dendritic cell maturation, activating macrophages, promoting T/B lymphocyte proliferation, regulating multiple signaling pathways, and stimulating the secretion of a broad range of cytokines (Song and Hu, 2009; Tiwari et al., 2018; Ma et al., 2022; Wan et al., 2022). Efforts are still needed to elucidate the underlying mechanisms.

Herein, we evaluate the immune-enhancing effect of several representative TCM, ingredients of TCM, and a compound herbal formula, including lentinan (polysaccharide), Panax notoginseng saponins (saponin), breviscapine (flavone), andrographolide (terpenoid), Astragali Radix, and kangai as adjuvants for the influenza vaccine. Because vaccination is by intramuscular injection, all TCM adjuvants in our study were injectable, approved in China, of controlled quality, safe, and stable, and injectable. The elicited immune response was characterized in terms of antibody production, splenocyte proliferation, splenic T cell differentiation, bone marrow dendritic cell maturation, and cytokine secretion. We found that TCM adjuvants can promote the innate and adaptive immune response of the influenza vaccine, and had the function of bidirectional regulation. In summary, Astragali Radix has a comprehensive effect on most of all evaluation indicators, which may be due to the polysaccharides, saponins, flavonoids and other constituents it contains (Chen et al., 2020). Our study is the first report to comprehensively assess the immune enhancing effects of TCM, its active ingredients, and its compounds on the influenza vaccine, suggesting that TCM is an attractive option for the development of effective and safe vaccine adjuvants.

## 2. Materials and methods

### 2.1. Reagents

Concanavalin A (Con A) and lipopolysaccharide (LPS) were purchased from ApexBio Technology Co., Ltd. and Sigma-Aldrich (Shanghai) Trading Co., Ltd., respectively. Mouse ELISA kits for IL-4 (20220111-20186A), IFN-γ (20220111-20140A), TNF-α (20220111-20852A), MCP-1 (20220111-20248A), IL-1 β (20220111-20174A), and IL-12 (20220111-20166A) were purchased from Shanghai Enzyme Linked Biotechnology Co., Ltd. The following flow cytometry antibodies were purchased: Brilliant Violet 605™ anti-Mouse CD45 (BioLegend), BV421 Rat anti-mouse CD25 (BD); BV510 Hamster anti-mouse CD3e (BD); mCD8-R208-APC (Sino Biological); mCD4-R711-FITC (Sino Biological); FITC anti-Mouse MHC-II (I-A/I-E) (eBioscience); BV421 Hamster anti-Mouse CD80 (BD); PE Rat anti-Mouse CD86 (BD); and BV786 Hamster ant-mouse CD11c (BD).

### 2.2. Influenza vaccine, virus, and TCM

The Quadrivalent Influenza Vaccine (split virion) was provided by Changchun Haijiya Biotechnology Co., Ltd., which included Influenza A and B virus strains constituents A/Brisbane/2/2018, A/Kansas/14/2017, B/Colorado/6/2017, and B/Phuket/3073/2013. Each influenza virus strain contains 15 μg of hemagglutinin per 0.1 mL of vaccine. The influenza virus strains were provided by Beijing Institute of Microbiology and Epidemiology and was propagated in chicken embryos and stored at −80°C until further use. Lentinan injection (1 mg per bottle) was purchased from Jiangsu Kangyuan Pharmaceutical Co., Ltd.; Sanqi Panax Notoginseng injection (150 mg per bottle) was purchased from Guangxi Wuzhou Pharmaceutical Co., Ltd.; Breviscapine injection (5 mL:20 mg) was purchased from Shenwei Pharmaceutical Group Co., Ltd.; Xiyanping injection (andrographolide sulfonated compound, 5 mL: 125 mg) was purchased from Jiangxi Qingfeng Pharmaceutical Co., Ltd.; and Astragalus (Huang Qi, 10 mL equivalent to 20 g of original medicine) injection was purchased from Heilongjiang Zhenbao Island Pharmaceutical Co., Ltd.; Kangai (20 mL) injection was purchased from Changbaishan Pharmaceutical Co., Ltd.

### 2.3. Animals and immunization protocol

**Figure 1:**
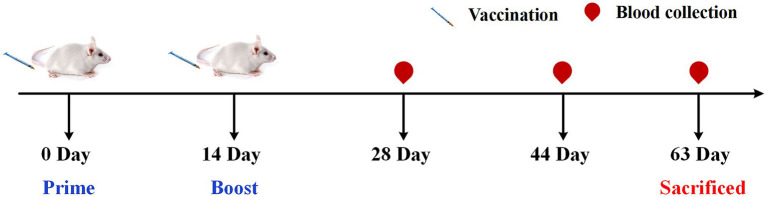
Schematic diagram of vaccination and sample collection.

Female BALB/c mice (8 weeks old) were purchased from SPF (Beijing) Biotechnology Co., Ltd. The mice were housed in an environment that met the National Standards of Laboratory Animal Requirements (GB 14925–2001) of China and all procedures were approved by the Animal Welfare and Ethics Committee of Beijing University of Chinese Medicine. The mice were randomly divided into nine groups (consisting of six mice each). The group immunized with the PBS solution was the control group, the mice in the other groups were immunized with 15 μg of hemagglutinin/mouse of Influenza vaccine alone (VA group) or together with various TCM adjuvants listed in Table 1. Dosage was determined according to the documented dosage of TCM as vaccine adjuvant and the clinical dose for human use (Sun et al., 2004; Aachoui et al., 2011; Yuan et al., 2012; Xing et al., 2016; Jin et al., 2020). Figure 1 shows a schematic diagram of vaccination and sample collection. The mice were immunized with primary and booster immunizations at days 0 and 14, respectively, each mouse was injected with 0.2 ml in the lateral part of the hind legs (0.1 mL per leg). The experiment ended 7 weeks after enhanced immunization (day 63), and serum, thymus, spleen, and bone marrow were collected for indicator detection. During which body weight and growth status were recorded and the original weight percentage of mice was calculated using the following equation: Original weight percentage (%) = weight of mice on day n/weight of mice on day 0 × 100. The thymus and spleen indices were calculated as follows: Index (mg per 10 g) = weight of the thymus or spleen (mg)/body weight (g) × 10.

**Table 1:** Animal grouping and vaccination dose.

Group number	Vaccine or TCM adjuvants for mouse immunization	Vaccine volume	TCM dosage and volume
A	PBS	/	/
B	Vaccine	0.1 mL	/
C	Vaccine+aluminum	0.1 mL	100 μg, 0.1 mL
D	Vaccine+lentinan	0.1 mL	100 μg, 0.1 mL
E	Vaccine+panax notoginseng saponins	0.1 mL	800 μg, 0.1 mL
F	Vaccine+breviscapine	0.1 mL	100 μg, 0.1 mL
G	Vaccine+andrographolide	0.1 mL	800 μg, 0.1 mL
H	Vaccine+Astragali Radix	0.1 mL	40 mg, 0.1 mL
I	Vaccine+kangai	0.1 mL	0.1 mL, 0.1 mL

### 2.4. Hemagglutination inhibition assay

Blood samples were collected and centrifuged to obtain the supernatant. The serum was treated with RDE (receptor destroying enzyme) at 56°C for 16 h and inactivated at 56°C for 1 h to remove the nonspecific statin. The serum was then serially diluted two times and incubated with the same volume of H1N1 virus hemagglutination titers for 30 min at room temperature, followed by 30 min with the addition of chicken red blood cells of the same volume. The HAI titers are expressed as the reciprocal of the highest serum dilution.

### 2.5. Splenic lymphocyte proliferation assay

Splenocytes were isolated from mice on D63 and spleen cell suspensions (1 × 10^6^ cells/mL) were prepared in RPMI 1640 medium supplemented with 10% FBS. The splenocytes were treated with Concavalin A (Con A-final concentration of Con A 5.0 μg/mL), lipopolysaccharide (LPS-final concentration of LPS 1.0 μg/mL) and incubated for 48 h at 37°C in an atmosphere of a 5% CO_2_. Four hours before the end of culture, 50 μL MTT solution (2 mg/mL) was added to the cell culture medium. At the end of the culture, plates were centrifuged, and the supernatant was discarded. A 150 μL volume of DMSO was added and avoiding light, and the plate was fully shaken until the blue particles were completely dissolved. The resulting absorbance was detected at 490 nm using a microplate reader. The proliferation activity of T and B lymphocytes was expressed using the stimulus index SI: SI = (experimental group OD490- blank group OD490)/(negative control OD490- blank group OD490). Each experiment was performed in triplicate (Zhang et al., 2017; He et al., 2022; Li Q. X. et al., 2022).

### 2.6. Enzyme-linked immunosorbent assay (ELISA) for cytokines

The concentrations of IL-4, IFN-γ, TNF-α, MCP-1, IL-1β, and IL-12 in the sera of mice were assayed using commercial ELISA kits according to the manufacturer’s instructions (Shanghai Enzyme-linked Biotechnology Co., Ltd.).

### 2.7. Lymphocyte immunophenotype and maturation of bone marrow dendritic cells by flow cytometry

Spleen cells and bone marrow cells were subcultured the bottom of in 96-U-well plates, and a premixed antibody mixture was added separately. Plates were incubated at 4°C in the dark for 20 min, add the washing solution was centrifuged and the cells washed twice, and resuspend for testing. The percentage of CD45^+^CD3^+^, CD3^+^CD4^+^, CD3^+^CD8^+^, and CD3^+^CD4^+^CD25^+^ in spleen cells, and the percentages of CD11c^+^MHC II^+^, CD11c^+^CD80^+^, CD11c^+^CD86^+^ in the bone marrow were measured by flow cytometry.

### 2.8. Statistical analysis

SPSS v20.0 software (IBM, New York, United States) was used to perform statistical analysis. All data were analyzed using one-way ANOVA followed by Tukey’s or Dunnett’s tests for multiple comparison, or a nonparametric Kruskal-Wallis test for group comparisons. Values were expressed as the mean ± standard deviation (SD). Statistical significance was considered at *p*-values less than 0.05. GraphPad Prism v7.0 (GraphPad, San Diego, United States) was used to plot the results.

## 3. Results

### 3.1. TCM-adjuvant had no side effects on the body weight of influenza vaccine immunized mice, and was consistent with the normal growth characteristics of mice

**Figure 2:**
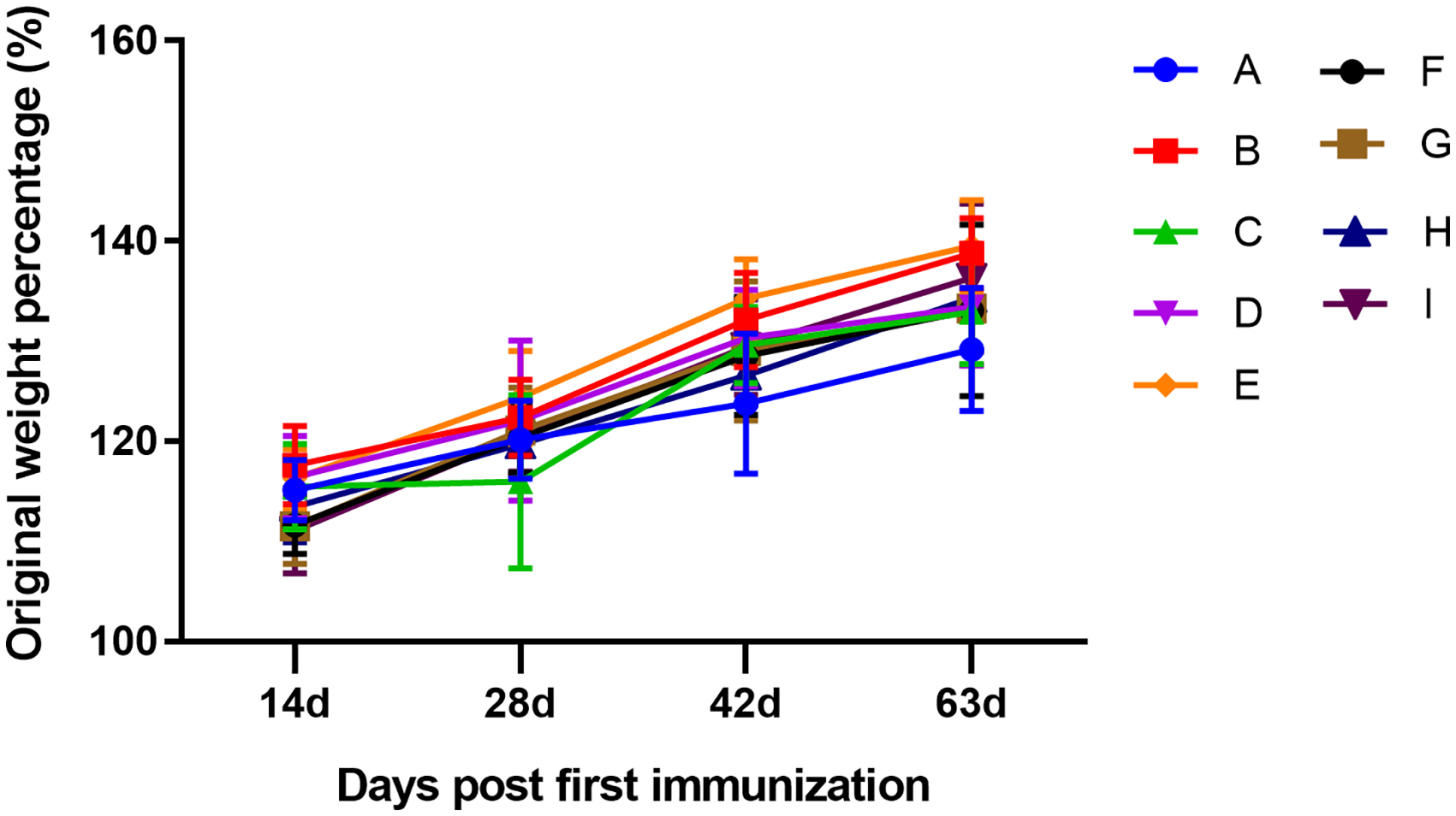
Dynamic changes in the percentage of original weight among groups. A, PBS group; B, influenza vaccination alone group; C, influenza vaccine and aluminum hydroxide co-immunized group; D, influenza vaccine and lentinan co-immunized group; E, influenza vaccine and panax notoginseng saponins co-immunized group; F, influenza vaccine and breviscapine co-immunized group; G, influenza vaccine and andrographolide co-immunized group; H, influenza vaccine and Astragali Radix co-immunized group; I, influenza vaccine and kangai co-immunized group. The letters in the following figures have the same meaning.

The body weight of the mice was recorded on days 0, 14, 28, 42, and 63, and the original weight percentage changes are shown in Figure 2. The body weight of the mice increased over time, and there were no significant differences among the groups, which was consistent with the normal growth characteristics of the mice and also indicated that the TCM adjuvant had no side effects on the mice, and no damage to the organs of the mice was observed during sampling.

### 3.2. TCM-adjuvant increased HAI antibody titers to influenza vaccine

**Figure 3:**
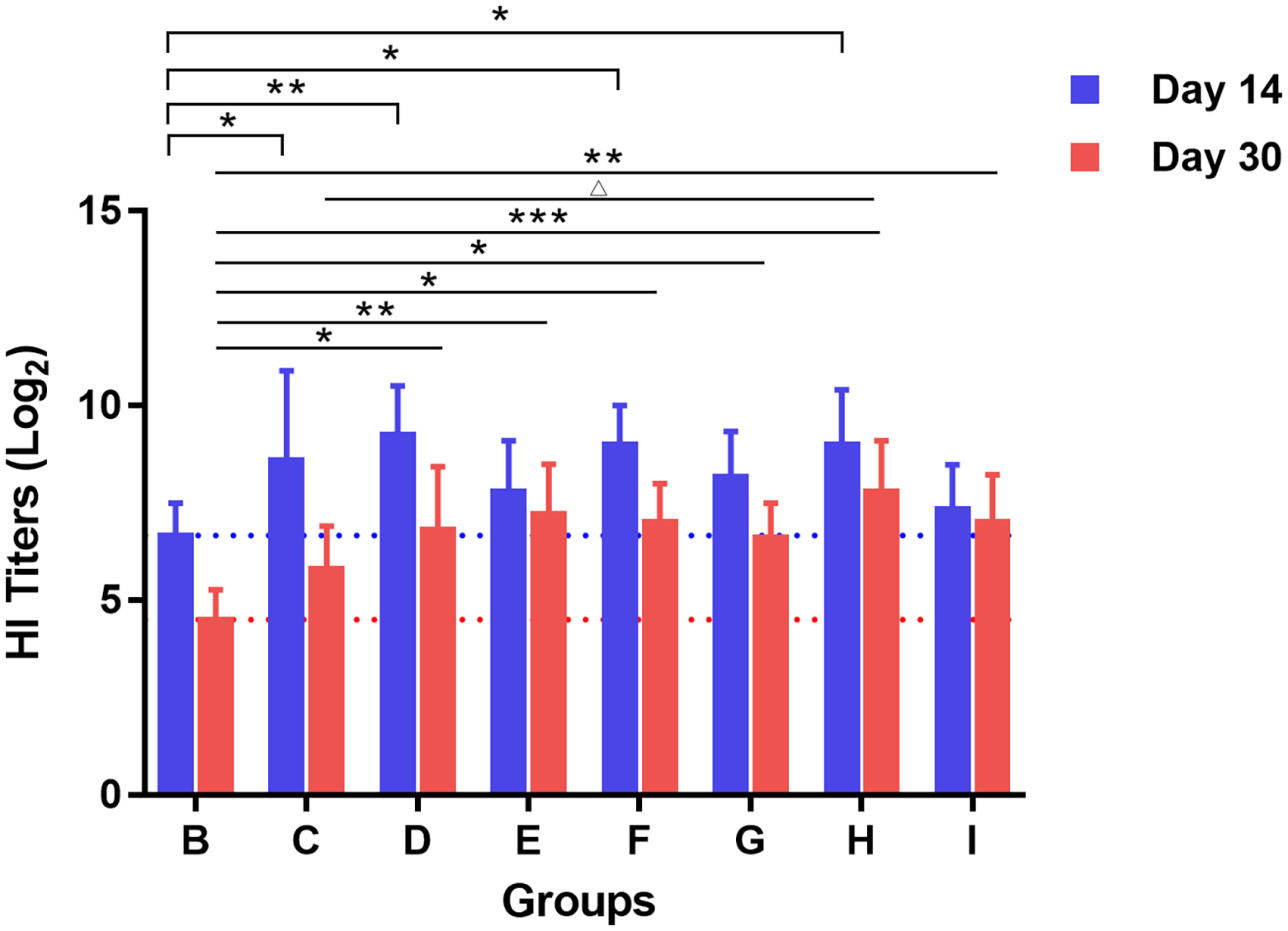
The response of HAI antibody titers to H1N1 virus measured by the hemagglutination inhibition assay on days 14 and 30 after booster vaccination. Each group was compared with the group given influenza vaccination alone (^*^p < 0.05, ^**^p < 0.01, ^***^p < 0.01), and influenza vaccine and Astragali Radix co-immunized group (^△^*p* < 0.05).

Serum from mice in each group was collected for HAI testing on days 14 and 30 after booster immunization. The results in Figure 3 showed that on day 14 after booster immunization, the HAI antibody titers in the TCM adjuvant groups increased to varying degrees compared to the vaccine immunization alone group, among which lentinan, breviscapine, and Astragali Radix had significant effects (*p* < 0.01, *p* < 0.05), and were slightly better than the aluminum adjuvant (*p* < 0.05). HAI antibody titers decreased on day 30 after booster immunization compared to day 14. For the intergroup, HAI antibody titers were still significantly higher in the TCM-adjuvant groups than in the immunized group with the vaccine alone (*p* < 0.01, *p* < 0.05, *p* < 0.001). Astragali Radix played a more prominent role than other TCM-adjuvant groups, and significantly higher than that of aluminum adjuvant (*p* < 0.05). These results demonstrated that the TCM adjuvant may enhance the response of the HAI antibody to influenza vaccines.

### 3.3. TCM-adjuvant regulated the immune organ index to influenza vaccine

**Figure 4:**
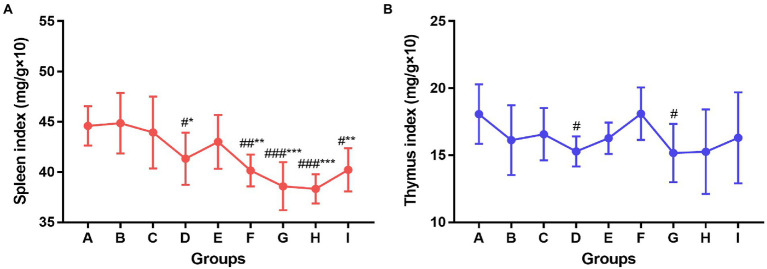
Changes of spleen index **(A)** and thymus index **(B)** among groups. Significant differences were observed between the PBS group and the vaccination groups (^#^*p* < 0.05, ^##^*p* < 0.01, ^###^*p* < 0.001), and between the influenza vaccination alone group and the other vaccination groups (^*^p < 0.05, ^**^p < 0.01, ^***^p < 0.001).

The spleen and thymus of the mice in each group were collected after the experiment and the organ index was calculated. As shown in Figure 4, compared to the control group and the influenza vaccination alone group, the spleen index of mice in the TCM adjuvant groups decreased significantly except for the Panax notoginseng saponins (*p* < 0.05, *p* < 0.01, *p* < 0.001). In combination with the HAI test, the TCM adjuvant increased antibody titers. TCM adjuvant had a bidirectional regulatory effect on immune organs. Specific results were determined in combination with other indicators. The thymus index results showed that the index decreased in the TCM-adjuvant group compared to the control group, which was consistent with changes in the spleen index, but the difference was not significant compared to the immunization group receiving only vaccine. Of the TCM adjuvants tested, lentinan and andrographolide decreased the thymus index significantly (*p* < 0.05), reflecting the regulatory effect of this TCM-adjuvant.

### 3.4. TCM-adjuvant promoted splenic lymphocyte proliferation to influenza vaccine

**Figure 5:**
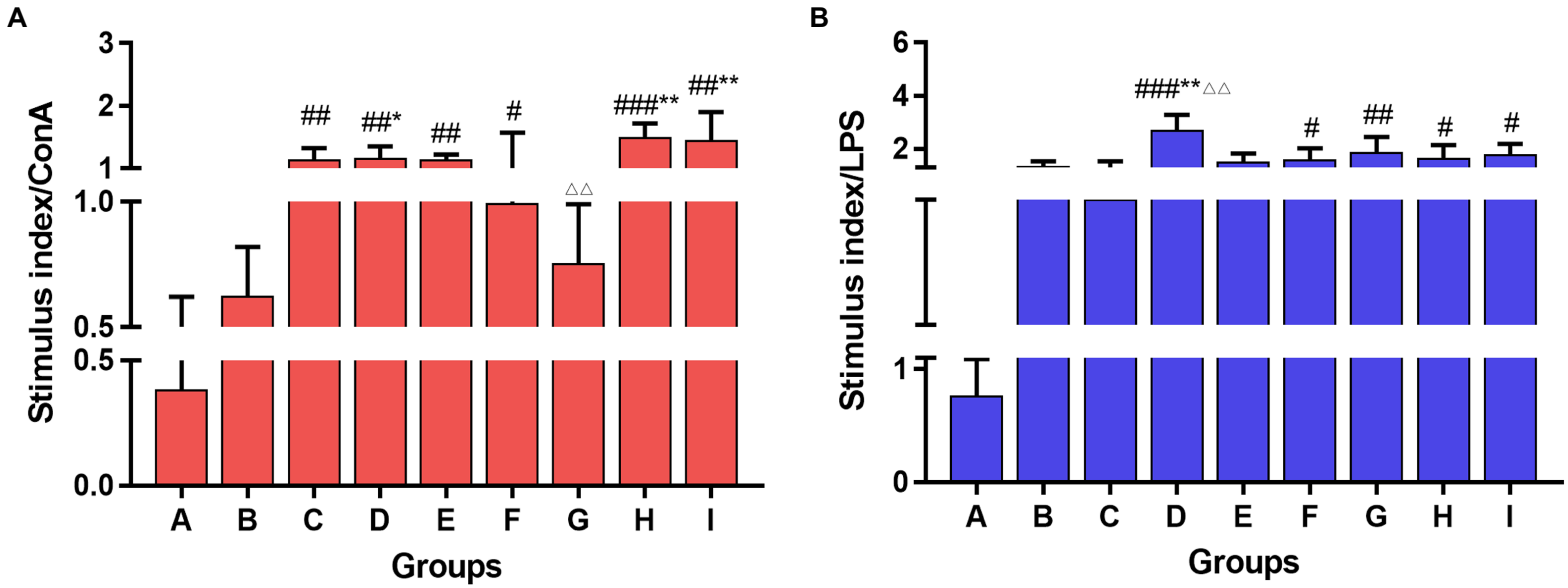
Lymphocyte proliferation of the spleen stimulated with Con A **(A)** and LPS **(B)** among groups. One-way ANOVA followed by Tukey’s multiple comparison test was conducted to compare two groups. Significant differences were defined between the PBS group and the vaccination groups (^#^*p* < 0.05, ^##^*p* < 0.01, ^###^*p* < 0.001), and between the influenza vaccination alone group and other vaccination groups (^*^p < 0.05, ^**^p < 0.01), and between the influenza vaccine and Astragali Radix co-immunized group and other TCM-adjuvant groups (^△△^*p* < 0.01).

Con A and LPS were used to stimulate T and B lymphocytes in the spleen of each group of mice, respectively, and cell proliferation was observed. The results are shown in Figure 5. The TCM adjuvant had a more marked role in promotion T cell proliferation, among which lentinan, Astragali Radix, and kangai had a significant increase (*p* < 0.05, *p* < 0.01) compared to the vaccination alone group, and the effect of Astragali Radix was significantly higher than that of andrographolide (*p* < 0.01). In terms of B cell proliferation, lentinan was more obvious than in those who were immunized with the vaccine alone (*p* < 0.01), and the other TCM adjuvant groups also showed a slight improvement.

### 3.5. TCM-adjuvant promoted the expression of the spleen T lymphocyte immunophenotype to influenza vaccine

**Figure 6:**
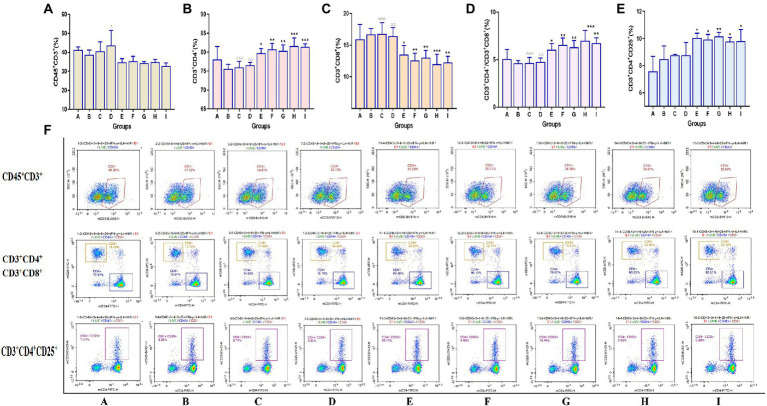
Changes in the expression of the Spleen T lymphocyte immunophenotype between groups detected by flow cytometry assay. **(A)** CD45^+^CD3^+^; **(B)** CD3^+^CD4^+^; **(C)** CD3^+^CD8^+^; **(D)** CD3^+^CD4^+^/CD3^+^CD8^+^; **(E)** CD3^+^CD4^+^CD25^+^; **(F)** representative flow cytometry plots of each detected T lymphocyte. A significant differences were defined between the influenza vaccination alone group and other vaccination groups (^*^p < 0.05, ^**^p < 0.01, ^***^p < 0.001), and between the influenza vaccine and Astragali Radix co-immunized group and other TCM-adjuvant groups (^△△^*p* < 0.01, ^△△△^*p* < 0.001).

Flow cytometry was used to detect the expression of the CD45^+^CD3^+^, CD3^+^CD4^+^, CD3^+^CD8^+^, and CD3^+^CD4^+^CD25^+^ subtypes of T lymphocytes in the spleen of mice, and the results are shown in Figure 6. Compared to mice in vaccination alone, TCM adjuvants can increase CD3^+^CD4^+^ content, and there were significant differences (*p* < 0.05, *p* < 0.01, *p* < 0.001) except for lentinan, which was also better than the aluminum adjuvant. The effect of Astragali Radix was significantly better than that of aluminum adjuvant and lentinan (*p* < 0.01, *p* < 0.001). The effect on CD3^+^CD8^+^ cell content showed an opposite trend, which was significantly reduced in the TCM adjuvant group (*p* < 0.05, *p* < 0.01, *p* < 0.001) except for lentinan, and the results of CD3^+^CD4^+^/CD3^+^CD8^+^ ratio was consistent with those of CD3^+^CD4^+^ (*p* < 0.05, *p* < 0.01, *p* < 0.001). It should be noted that the CD3^+^CD4^+^CD25^+^ content in the TCM adjuvant group increased (*p* < 0.05, *p* < 0.01), which is a T-regulatory cell and has the immunosuppressive effect (Yang et al., 2018), once again confirmed speculation in the organ index results. TCM adjuvants may have a bidirectional regulatory effect, reflecting the advantage of TCM as a vaccine adjuvant in immune regulation.

### 3.6. TCM-adjuvant promoted the activation of bone marrow dendritic cells to influenza vaccine

**Figure 7:**
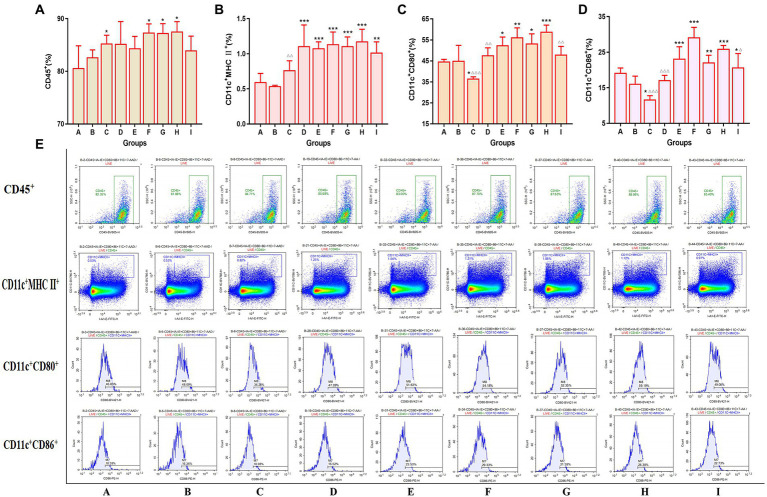
Changes in the expression of bone marrow co-stimulatory signaling molecules across the indicated groups as detected by flow cytometry. **(A)** CD45^+^; **(B)** CD11c^+^MHC II^+^; **(C)** CD11c^+^CD80^+^; **(D)** CD11c^+^CD86^+^; **(E)** representative flow cytometry plots of each detected co-stimulatory signaling molecules. Significant differences were defined between the influenza vaccination alone group and other vaccination groups (^*^p < 0.05, ^**^p < 0.01, ^***^p < 0.001), and between the influenza vaccine and Astragali Radix co-immunized group and other TCM-adjuvant groups (^△^*p* < 0.05, ^△△^*p* < 0.01, ^△△△^*p* < 0.001).

The expression of costimulatory signal molecules of dendritic cells was detected in the bone marrow of mice by the flow cytometry. The results are shown in Figure 7. Compared to the mice in the vaccination alone group, all of the TCM adjuvant groups could significantly increase the expression of CD11c^+^MHC II^+^ signal molecules (*p* < 0.01, *p* < 0.001), and the effects were better than those of aluminum adjuvant. In addition to the lentinan and Kangai compounds, the TCM adjuvant groups could significantly increase the expression of CD11c^+^CD80^+^ (*p* < 0.05, *p* < 0.01, *p* < 0.001), and except for lentinan, the expression of CD11c^+^CD86^+^ in the TCM adjuvant groups increased significantly (*p* < 0.05, *p* < 0.01, *p* < 0.001). The content of CD11c^+^CD80^+^ and CD11c^+^CD86^+^ in the aluminum adjuvant group decreased significantly (*p* < 0.05). Among which the effect of Astragali Radix was significantly better than that of the aluminum, lentinan and Kangai compounds (*p* < 0.05, *p* < 0.01, *p* < 0.001). The results suggest that TCM adjuvants may play a role in immune promotion in vaccines by enhancing the co-stimulation signal molecules on the surface of dendritic cells and promoting their maturation.

### 3.7. TCM-adjuvant regulated the secretion of cytokines to influenza vaccine

**Figure 8:**
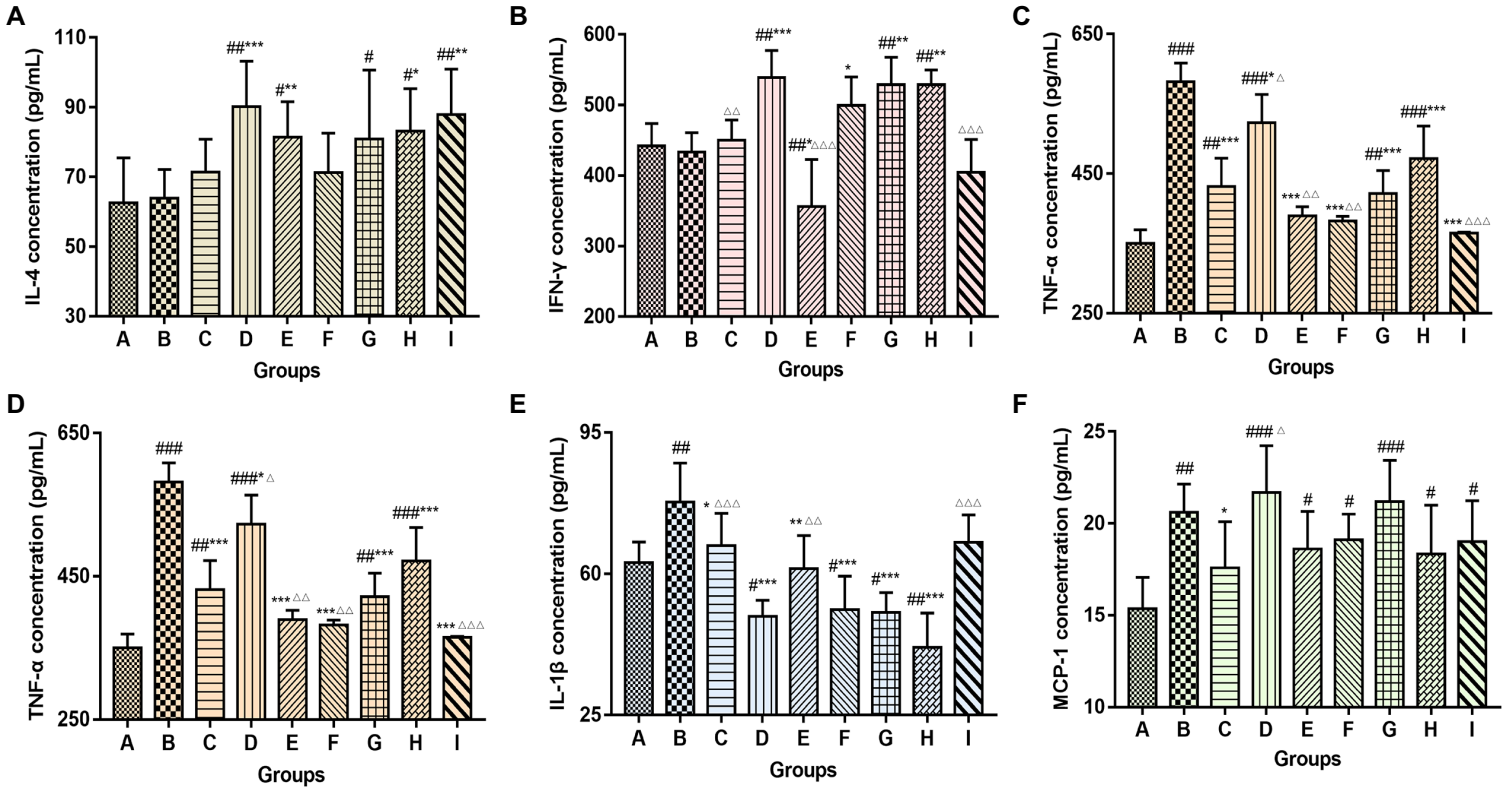
Changes in serum cytokine content (**A**, IL-4; **B**, IFN-γ; **C**, TNF-α; **D**, IL-12; **E**, IL-1β; **F**, MCP-1) among groups. The significant differences were defined between the PBS group and the vaccination groups (^#^*p* < 0.05, ^##^*p* < 0.01, ^###^*p* < 0.001), and between the influenza vaccination alone group and the other vaccination groups (^*^p < 0.05, ^**^p < 0.01, ^***^p < 0.001), and between the influenza vaccine and Astragali Radix co-immunized group and other TCM-adjuvant groups (^△^*p* < 0.05, ^△△^*p* < 0.01, ^△△△^*p* < 0.001).

Mice serum was collected at the end of the experiment to detect cytokine changes by ELISA, including the levels of Th1 cytokines IFN-γ, TNF-α; Th2 cytokines IL-4; and other cytokines IL-12, IL-1β, and MCP-1 (Figure 8). Compared to the control group and the vaccination-alone group, TCM adjuvants can increase the content of IL-4, IFN-γ and MCP-1 (*p* < 0.05, *p* < 0.01, *p* < 0.001), and decrease the content of TNF-α, IL-12 and IL-1β (*p* < 0.05, *p* < 0.01, *p* < 0.001). Overall, lentinan, andrographolide, and Astragali Radix (*p* < 0.05, *p* < 0.01, *p* < 0.001) had more significant effects. The results suggest that TCM adjuvants can not only regulate the Th1 immune response, but also the Th2 immune response, and may have the advantage of bidirectional regulation without excessive expression of cytokines.

**Figure 9:**
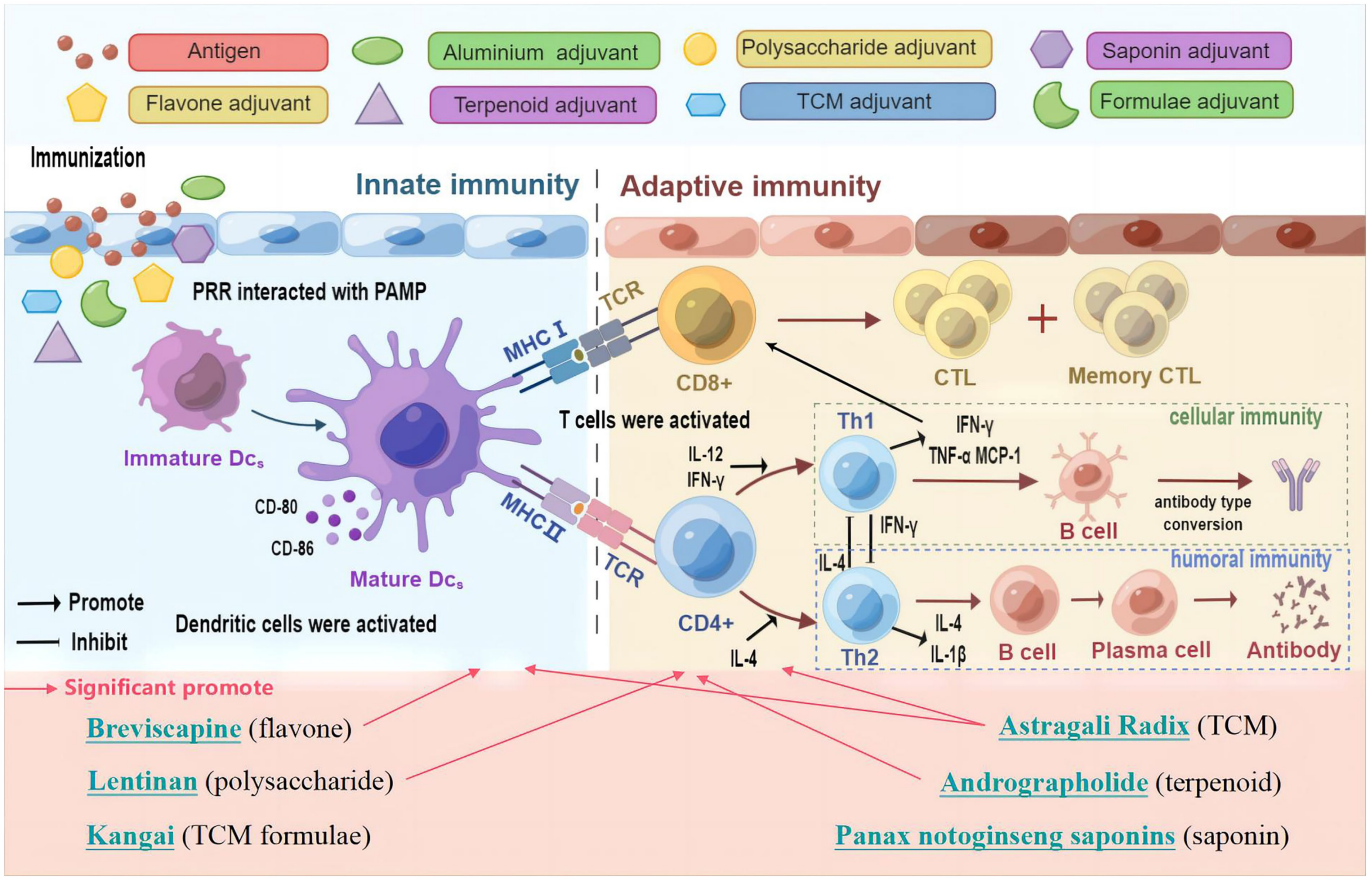
Schematic diagram of the mechanisms induced by TCM and its active ingredients to improve influenza vaccine immune responses. Figure drawn using Figdraw.

## 4. Discussion

The influenza vaccine is an effective approach to tackle influenza virus infection (Laupeze et al., 2021). However, there is still an unmet need to improve the recipient’s immune response to the influenza vaccine in many cases, like the drifted or shifted influenza strains, weak immunogenicity of some influenza vaccines, such as split and subunit vaccines, few licensed adjuvants for humans, and failure to induce the cytotoxic T cell response of adjuvants, and so on (Wei et al., 2020; Nagashima and Mousa, 2021; Zhu et al., 2021). In this study, we performed a comprehensive comparison of multiple TCM and its ingredients, including lentinan, Panax notoginseng saponins, breviscapine, andrographolide, Astragali Radix, kangai, when formulated with influenza vaccine to determine whether these were able to elicit a stronger immune response.

To be consistent with the intramuscular injection vaccine immunization method, the TCM adjuvants used in our study were all injections, having strict quality control standards, including the standards of the Ministry of Health of the People’s Republic of China or the standards of the National Medical Products Administration. Lentinan for injection should contain 90.0–115.0% of the labeled amount (NO. WS_1_-(X-152)-2004Z). The total saponins of Panax notoginseng in each of Xueshuantong injection were calculated as ginsenoside Rg_1_ (C_42_H_72_O_14_), which should be 90.0–110.0% of the labeled amount (NO. WS_3_-B-3829-98). The injection of breviscapine should be 95.0–105.0% of the amount labeled, calculated with breviscapine B (C_21_H_18_O_12_) (NO. WS_3_-B-3822-98). Each injection of Xiyanping contains andrographolide sulfonate, which should be 90.0–110.0% of the labeled amount, calculated by sodium andrographolide sulfonate (C_20_H_29_O_5_·SO_3_Na) (NO. WS-10863(ZD-0863)-2002). Astragaloside IV (C_41_H_68_O_14_) should not be less than 0.08 mg per 1 mL (equivalent to 2 g of original medicinal materials) of injection of Astragali Radix (NO. WS_3_-B-3335-98). Kangai injection contains ginseng per 1 mL, which should not be less than 0.1 mg in the total amount of ginsenoside Rg_1_ (C_42_H_72_O_14_) and Re (C_48_H_82_O_18_), and 9.0–11.0 mg in the total amount of oxymatrine (C_15_H_24_N_2_O_2_) per 1 mL [No. WS-11222(ZD-1222)-2002]. This provides preliminary safety assurance for future use in combination with vaccines.

Vaccine antigens or adjuvants can be recognized as pathogen-associated molecular patterns (PAMPs) by pattern recognition receptors (PRRs) on the surface of innate immune cells, and trigger the innate immune response, and then by activating the downstream signaling, they promote cytokine secretion and regulates the adaptive immune response (Mahla et al., 2013; Zhu et al., 2021). Thus, innate and adaptive immunity play a crucial role in the viral vaccination (Hosseini et al., 2020; Li C. F. et al., 2022). Dendritic cells (DC) are typical innate immunity cell types and are the most potent antigen presenting cells (APCs). Immature or resting cDC have a lower expression of major histocompatibility complex (MHC) class II molecules and co-stimulatory molecules (e.g., CD80, CD86). cDCs undergoes maturation in response to activation by vaccination and infection, and lead to the upregulation of MHC class II and co-stimulatory molecules at the cell surface (Dalod et al., 2014). Mature DCs migrates to lymph nodes to interact with naive T cells (Russell, 2008), then naive CD4^+^ and CD8^+^ T cells can be activated, respectively, through the recognition and binding of the T cell receptor (TCR) on their surface to MHC II and MHC I on DCs, and by releasing costimulatory molecules and cytokines (such as IL-12) (Anderson et al., 2018). Upon activation, lymphocytes proliferate, an adaptive immune response is followed and forms an antigen-specific immune response through the production of cytokines by CD4^+^ T cells, the cytotoxic activity of CD8^+^ T cells or the production of antibodies by B cells (Walsh and Mills, 2013). Furthermore, CD4^+^ T cells as helper T cells (Th) may help activate CD8^+^ T cells and T-dependent B cells. A proportion of effector lymphocytes turns into memory T or B cells, and are used to resist reinfection with the same antigen (Zander et al., 2019; Eisenbarth et al., 2021). Therefore, prompting DC maturation can lead to the antigen processing and presentation, migration and the priming of adaptive immune responses based on B and T cells (Zepeda-Cervantes et al., 2020). It has been found that the adjuvant influenza vaccine formula could induce the phenotypic and functional maturation of DCs (Athale et al., 2017; Shirai et al., 2020; Yin et al., 2021). Influenza vaccine-mediated protection is dependent on CD4^+^ T cells, influenza-specific CD4^+^ memory T cells, which have the potential to overcome the poor immunogenicity of vaccines, which is needed for early antibody production and CD8^+^ T cell recall responses (Valkenburg et al., 2018; DiPiazza et al., 2019). Therefore, CD4^+^ and CD8^+^ T cell responses are broadly used to evaluate vaccine immune responses (Liao et al., 2020). In this study, mice immunized with the TCM-adjuvanted influenza vaccine exhibited significantly higher expression of CD11c^+^MHC II^+^, CD11c^+^CD80^+^, CD11c^+^CD86^+^, and CD3^+^CD4^+^, and have a higher proliferation index of T and B cell, compared with mice receiving vaccination alone, indicating that TCM may promote the maturation of DCs and activate innate and adaptive immunity in the immune response of the influenza vaccine. In particular, we observed a decrease in CD3^+^CD8^+^ levels and an increase in CD3^+^CD4^+^CD25^+^ levels, which may be attributed to bidirectional regulation by TCM.

To orchestrate a variety of adaptive immune responses, CD4^+^ T cells differentiate toward Th1, Th2, Th17, follicular T helper (Tfh), or regulatory T cells (Treg) upon activation (Butcher and Zhu, 2021). Among them, Th1/Th2 responses play an important role in vaccination and are the main regulatory targets of vaccine adjuvants (Chung et al., 2022; Kumar et al., 2022). Emulsion adjuvants such as MF59 and AS03 can induce a mixed Th1/Th2 T cell response (O'Hagan et al., 2021). QS-21, a saponin adjuvant could induce balanced Th1/Th2 immunity (Wang, 2021). By promoting the Th1/Th2 response, the adjuvanticity of polysaccharides for the influenza vaccine was improved (Li Q. X. et al., 2022). The most widely used aluminum adjuvant is unable to effectively induce Th1 cellular immunity (Wang Z. B. et al., 2022), which was also demonstrated in our study. Cytokines play a pivotal role in the Th immune response (Dong, 2021). Th1 cell differentiation is promoted by IL-12 (Watford et al., 2003), which produces IFN-γ, TNF-α, and IL-2, and can activate CD8^+^ cytotoxic T lymphocytes (CTLs), promote the production of IgG antibodies by B cells, and mediate cellular immunity. Th2 cells are mainly induced by IL-4 (Walker and McKenzie, 2018), and secrete IL-4, IL-5, and IL-13, which mediate humoral immunity. IL-4 could convert IgG antibodies to IgE in B cells (Berger, 2000). IL-1β is an inflammatory cytokine, which can induce potent T cell responses and tissue-resident memory T cells, leading to full protection against influenza virus as a vaccine adjuvant (Lapuente et al., 2018; Van den Eeckhout et al., 2020). Monocyte chemoattractant protein 1 (MCP-1) can attract or enhance the expression of other inflammatory cells (Singh et al., 2021), and with an enhancement level in the influenza vaccination process (Cao et al., 2016; Nakayama et al., 2018). Our study demonstrated that TCM and its active ingredients can promote Th1 and Th2 immune responses of influenza vaccines without excessive cytokine production, which is potent for vaccine adjuvants.

In summary, Astragali Radix has a more prominent immune-promoting effect on the influenza vaccine than other TCM adjuvants, and promotes both innate and adaptive immune responses, which may be related to Astragali Radix containing polysaccharides, saponins, and other components, with multiple immunomodulatory activities (Chen et al., 2020); To further investigate the efficacy of Astragali Radix as an influenza vaccine adjuvant, we conducted an influenza virus challenge experiment. The results showed that Astragali Radix could prolong the survival time of mice infected with influenza virus and was related to the dose (Supplementary Figure 1). In the experiment, the high dose of Astragali Radix did not show obvious protective advantage against the virus, which may be related to the excessive virus of challenge, leading to the death of all mice before the observation period of 14 days. Further studies should be carried out by optimizing the dose of the virus and Astragali Radix in future experiments. Lentinan exerts obvious regulatory effects on the HAI antibody titer, LPS-induced B cell proliferation and cytokines, which is consistent with the results reported in the literature indicating that lentinan can regulate adaptive immunity as a vaccine adjuvant (Xing et al., 2016; Jin et al., 2020). Breviscapine had a stronger effect in promoting bone marrow cell maturation and tended to regulate innate immunity. This is different from the conventional understanding of the use of breviscapine in cardiovascular disease (Min et al., 2020), but it has an anti-inflammatory effect, and inflammation is closely related to immunity, so the relationship between breviscapine and immunity can be further studied from the perspective of inflammation. The ability of andrographolide to regulate the organ index and spleen lymphocyte subtypes was more prominent and tended to regulate adaptive immunity. This is consistent with a literature report indicating that Xiyanping can enhance T lymphocyte activity (Li et al., 2018). Of note, the enhancing effect of Sanqi Panax Notoginseng and Kangai injection on innate and adaptive immunity was weaker than that of other TCM adjuvants. Therefore, different TCM adjuvants can be selected based on the requirements of different vaccines for immune enhancement in future applications.

While TCM and its active ingredients show promising potential as vaccine adjuvants, the above evaluation was performed as a preliminary experiment. It is also worth noting that, as with any study, our study had some limitations. Antigen and TCM adjuvants were compared in a single dose, and further studies should conduct multidose comparative analyses. It is also necessary to evaluate the adjuvant effect of TCM against other vaccine antigens and to extend studies to additional animal models, such as the ferret. Furthermore, the safety of TCMs should be further assessed in conjunction with vaccination, although these TCMs have been approved for human injection. Further studies are also required to investigate the molecular mechanisms of TCM adjuvant function.

## 5. Conclusion

The results of the present study demonstrate that Astragali Radix, lentinan and other TCM and its active ingredients could enhance the immune effect of the influenza vaccine without triggering an excessive immune response, characterized of balance and bidirectional regulation. In summary, Astragali Radix had a more comprehensive effect on vaccine immunity, including the regulation of both innate and adaptive immunity, while other TCM adjuvants had a regulatory effect on innate immunity or adaptive immunity (The schematic diagram of the mechanism is shown in Figure 9). Considering the feasibility and availability of TCM adjuvants, TCM and its active ingredients may represent a novel approach to potentiate the efficacy of influenza vaccines during pandemic outbreaks.

## Data availability statement

The raw data supporting the conclusions of this article will be made available by the authors, without undue reservation.

## Ethics statement

The animal study was reviewed and approved by the Experimental Animal Ethics Committee of Beijing University of Chinese Medicine (No. BUCM-4-2021072302-3034).

## Author contributions

LW and JZ designed and supervised the experiments. DZ, XC, RL, LT, YC, RT, XL, YC, and JH conducted the experiments. DZ wrote the original manuscript. WH, LW, and JZ reviewed and edited the manuscript. All authors have read and agreed to the published version of the manuscript.

## Funding

This work was supported by the National Key R&D Program of China (Nos. 2018YFC1706800, 2019YFC1200604, 2021YFD1800504, and 2022YFC2305005).

## Conflict of interest

The authors declare that the research was conducted in the absence of any commercial or financial relationships that could be construed as a potential conflict of interest.

## Publisher’s note

All claims expressed in this article are solely those of the authors and do not necessarily represent those of their affiliated organizations, or those of the publisher, the editors and the reviewers. Any product that may be evaluated in this article, or claim that may be made by its manufacturer, is not guaranteed or endorsed by the publisher.
