# Supplementary material for: Improvement influenza vaccine immune responses with traditional Chinese medicine and its active ingredients
Source: Front Microbiol. 2023 Mar 7;14:1111886. doi: 10.3389/fmicb.2023.1111886 (PMC10027775; doi:10.3389/fmicb.2023.1111886)
Supplement: Supplementary file 1 [file Data_Sheet_1.doc]

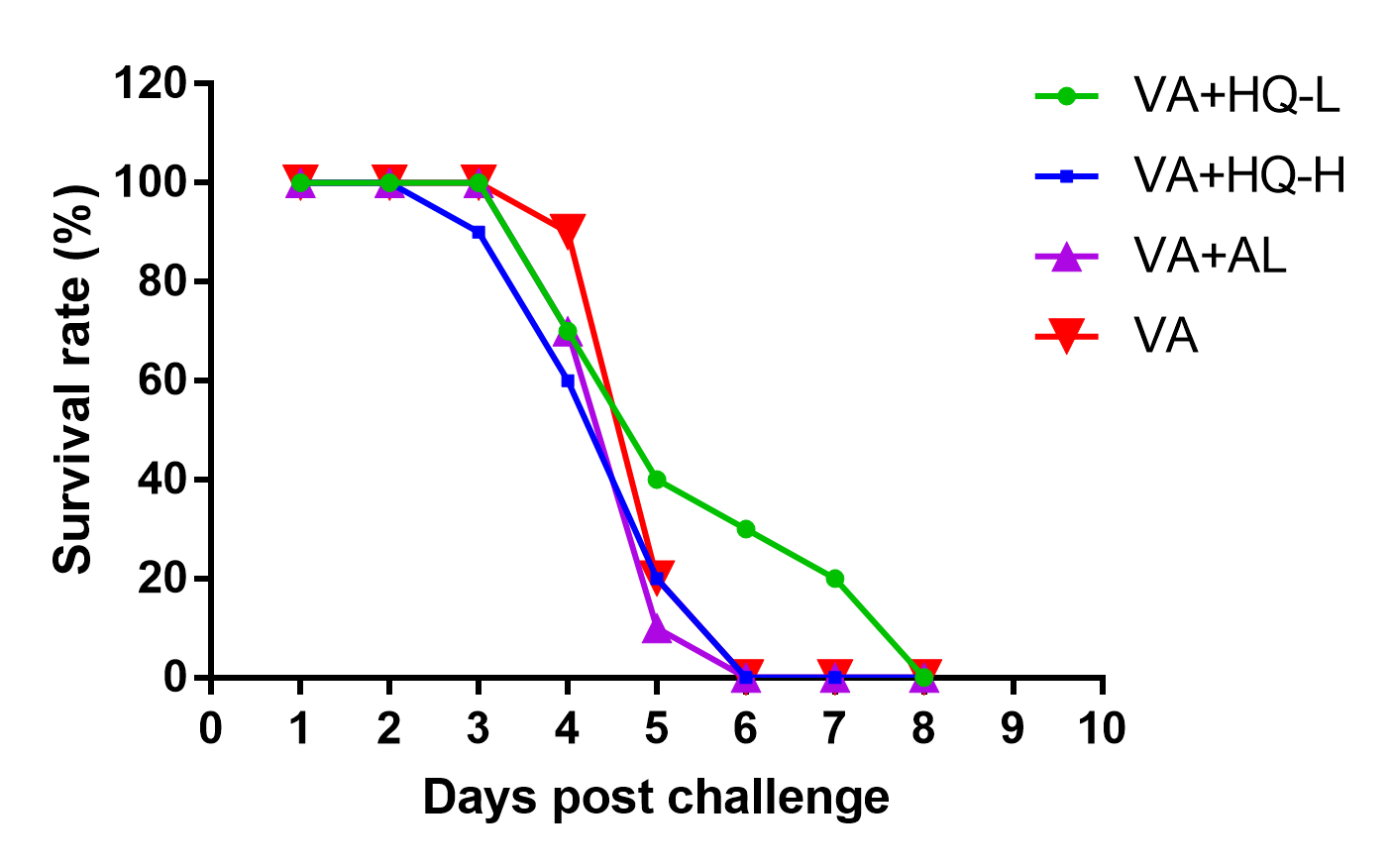


**Supplementary Figure 1. Survival rate of mice after the challenge with influenza PR8 virus.** 15 μg of hemagglutinin/mouse of Influenza vaccine alone (VA group) or together with aluminum hydroxide 100 μg/mouse (VA+AL group), Astragali Radix 20 mg/mouse (VA+HQ-L group), Astragali Radix 40 mg/mouse (VA+HQ-H group), were injected into mice via [intramuscular](javascript:;) route twice interval 2 weeks, with 10 mice in each group. 14 days after booster immunization, the mice were challenged with influenza A/PR/8/34 (H1N1) virus (5 LD_50_) by intranasally with 50 μL per mouse. Mice that lost > 30% body weight were euthanized.
